# Psychometric Properties of the Persian Version of Type D Personality Scale (DS14)

**Published:** 2011

**Authors:** Reza Bagherian, Hadi Bahrami Ehsan

**Affiliations:** 1 Assistant Professor, Behavioral Sciences Research Center and Department of Psychiatry, School of Medicine, Isfahan University of Medical Sciences, Isfahan, IRAN.; 2 Associate Professor, Department of psychology, University of Tehran, Tehran, Iran.

**Keywords:** Myocardial Infarction, Negative affectivity, Psychometric Properties, Social inhibition, Type D Personality Scale

## Abstract

**Objective:** Type D personality is based on negative affectivity (NA) and social inhibition (SI). The purpose of this study was to examine the reliability and validity of the Persian version of 14-item Type D personality scale (DS14).

**Methods:** The study included 191 normal participants and 176 patients with myocardial infarction (MI). They all filled out the Persian version of DS14, containing 7-item NA and SI subscales; 71 normal subjects completed the neuroticism and extroversion subscales of Eysenck questionnaire. Besides, 71 participants filled out the DS14 twice over a 2-month period.

**Results:** In this study, 35.8 % of MI patients and 24.6% of healthy people were classified as Type D. Factor analysis of Persian version of DS14 yielded the two-factor structure; all of the NA and SI items loaded between 0.65 to 0.80 in patients and 0.48 to 0.79 in healthy people on their corresponding factor. Test–retest stability of the NA and the SI subscales were 0.86 and 0.77 respectively over a 2-month period. High internal consistency of Cronbach's alpha coefficient has been found to be 0.84 in patients and 0.87 in healthy people for the NA sub-scale and 0.86 in patients and 0.75 in healthy people for the SI sub-scale. The construct validity of NA and SI sub scales were confirmed against neuroticism (r = 0.65) and extroversion (r = -0.62) subscales of Eysenck questionnaire respectively.

**Conclusion:** Persian version of DS14 is an acceptable, reliable and valid measure of dimensions of Type D personality.

## Introduction

Personality is an important mediator of chronic stress. However, the controversy over Type A behavior made it unfashionable to include global traits in coronary artery disease (CAD) research (1,2,3). Subsequent research mainly focused on specific traits such as hostility and anger (4). Recently, a new personality construct, the Type D or ‘distressed’ personality, has been proposed (5). This construct is a result of an investigation of coping styles in men with coronary heart disease (6). Contrary to Type A behavior construct, Type D personality emerged from existing personality theory and empirical evidence (5). The taxonomy is based on two global and stable personality traits including negative affectivity (NA) and social inhibition (SI) (5,6). NA refers to the tendency to experience negative emotions across time/ situations (1,7). High-NA individuals experience more feelings of dysphoria, anxiety, and irritability; have a negative view of self; and scan the world for signs of impending trouble (1). SI refers to the tendency to inhibit the expression of emotions/behaviors in social interactions to avoid being disapproved by others (1,5). High-SI individuals tend to feel inhibited, tense, and insecure when they are with others (1). Individuals who are high in both NA and SI have a distressed or Type D personality and are vulnerable to chronic distress (1). Type D patients are at an increased risk for a wide range of adverse health outcomes (5). Physiological hyperreactivity (8), immune activation (9), and inadequate response to cardiac treatment (10,11) are mechanisms that may explain this detrimental effect of Type D. Finally, Type D patients are at risk for clustering of psychologic risk factors including depression, anxiety, and irritability, and low levels of self-esteem, well-being, and positive affect (12).

The Type D Scale-14 (DS14) was specifically developed to evaluate NA, SI, and Type D in a reliable and standardized method with a little burden on the respondents (1). Items of the DS14 have been derived from its predecessor, the DS16 (13), but it also consists of new items developed to promote the assessment of NA and SI (1).

A recent study conducted by Denollet on the psychometric properties of the DS14 clearly indicates that it is a brief and well-performed questionnaire in assessing both Type D dimensions. Denollet found that the NA and SI scales were internally consistent (α = 0.88/0.86; N = 3678) and stable over a 3-month period (test–retest r = 0.72/0.82). NA correlated positively with neuroticism (r = 0.68); SI correlated negatively with extraversion (r=_0.59/_0.65). Scale-level factor analysis confirmed the construct validity of the DS14 against the NEO-FFI (1). Pedersen and Denollet examined validity of the Type D personality construct in Danish post-MI patients. Their study confirmed the two-factor structure of the Type D personality scale-16 (DS16) and the internal consistency of the NA (a = .83) and SI (a = .76) subscales. The construct validity of the DS16 was confirmed against scales that measure similar constructs (2). The construct was developed in Belgian cardiac patients, but little is known about its applicability in other nationalities.

Up to now, there has been no study of validity and applicability of DS14 in Iranian sample. This study was performed: 1) to provide and examine the reliability and validity of Persian version of DS14, 2) to determine the prevalence of Type D personality in the patients with MI.

## Materials and Methods

*Translation:*

The 'forward-backward' procedure was applied to translate the DS14 from English into Persian (Iranian language). A psychologist, Ph.D, with aid of a Ph.D student of English language translated the questionnaire into Persian and this was backward translated into English by a health psychologist and a professional translator. There were some problematic terms which were culturally adapted with the aid of someone being bilingual and the final version was developed after a consensus by ten psychologists and psychiatrists.

*Sample, data collection:*

The sample included 176 consecutive patients with MI admitted to the CCU wards of nine hospitals in Isfahan, Iran, over a 5-month period (148 men and 28 women; mean age: 56 years; range: 32–84 years and 191 Healthy subjects who were randomly selected from Tehran university students (N=191; 96 men and 95 women; mean age: 24.6 years; range: 19–47 years) and 71 subjects of general population visiting patients hospitalized in the university affiliated hospitals in Isfahan (N=71; 30 men and 41 women; mean age: 31.1 years; range: 19–63 years). 

MI Patients were eligible if they met the criteria of MI diagnosis. On the other hand, they were excluded if they had a life expectancy of less than 1 year because of comorbid noncardiac disease (e.g., malignancies), had poor cognitive functions, had major psychiatric disorders, were unable to speak or read Persian, or had visual or auditory problems that precluded their participation.

The final draft of the Persian version of DS14 was completed by the patients and healthy people. They all filled out the Persian version of DS14, containing 7-item NA and SI subscales. Seventy-one subjects of the general population completed the neuroticism and extroversion subscales of Eysenck’s questionnaire. Besides, these 71 participants filled out the DS14 twice over a 2-month period.

*Questionnaire:*

*The Persian version of Type D personality scale:* The DS14 include two subscales, NA and SI, each of which contains 7 items. These items are answered on a five-point Likert scale from 0 (false) to 4 (true) giving maximum scores of 28 for NA and SI. A predetermined cut-off of ≥ 10 on both subscales is used to determine those with a Type D personality (1,12). Examples of items measuring NA are ‘I often make a fuss of unimportant things’, and ‘I often feel unhappy’. Furthermore, ‘I often feel inhibited in social interactions’, and ‘I find it hard to start a conversation’ are examples of items of the SI subscale. According to an investigation into psychometric properties of DS14 conducted by Denollet, NA and SI scales were internally consistent (α = 0.88/0.86; N = 3678) and stable over a 3-month period (test–retest r = 0.72/0.82). NA correlated positively with neuroticism (r = 0.68); SI correlated negatively with extraversion (r=_0.59/_0.65). Scale-level factor analysis confirmed the construct validity of the DS14 against the NEO-FFI (1).

*The Persian version of* *Eysenck’s* *questionnaire:* Iranaian Eysenck’s questionnaire is a valid, reliable and common questionnaire in assessing personality in Iranian society. The questions of Eysenck’s questionnaire cover the personality aspects of Eysenck’s hypothesis and have content validity (14).

*Statistical Analyses:*

There were almost no missing values. Principal components analysis (varimax rotation) was used to examine the internal–structural validity, ie, the ability of items to reflect the personality traits of NA and SI. A scree plot was used to determine principal components to retain. Cronbach’s alpha coefficient was used to examine the internal consistency of the DS14. Pearson’s correlation was used to examine the internal consistency of items of both subscales and the construct validity of the Persian DS14 against the Iranaian Eysenck’s questionnaire. Test–retest correlations over a 2-month period were calculated for the DS14 personality.

## Results

The sample included 176 consecutive patients with MI [148 men and 28 women; mean age: 56 (S.D.=10.053) years; range: 32–84 years, 88%:married] and 191 healthy subjects from the general population (96 men and 95 women; mean age: 31.1 years; range: 19–63 years). Using a cutoff of 10, 63 patients (35.8 %) were classified as Type D personality and of 191 healthy people, 46 (%24.6) were characterized as type D personality.

**Figure 1:**
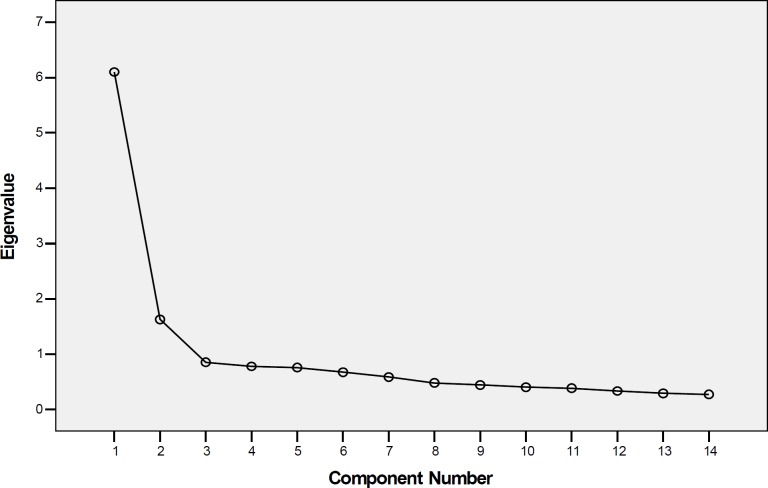
Scree plot showing the eigenvalues of the factors representing the items from the DS14 for patients.

**Figure 2:**
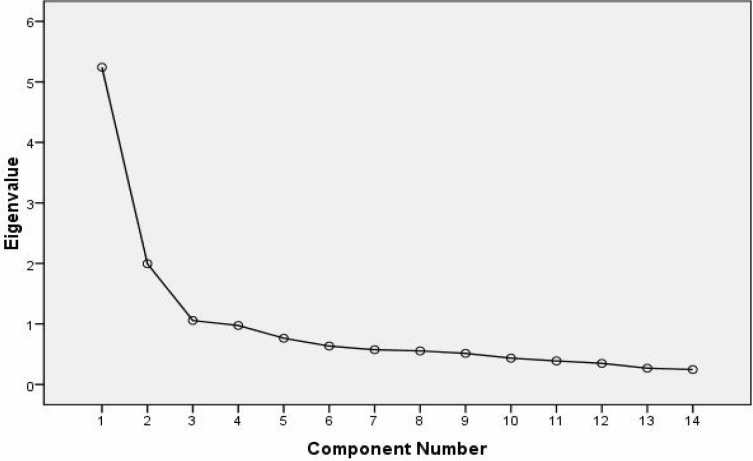
Scree plot showing the eigenvalues of the factors representing the items from the DS14 for healthy people

Factor analysis confirmed a structural validity of the NA and SI items of the Persian DS14. Eigenvalue and scree plot criteria indicated 2 dominant personality domains (Figures 1 and 2). All of the 7 NA items and 7 SI items had a loading ranging from 0.65 to 0.80 for patients’ group and 0.52 to 0.79 for healthy group on their corresponding trait factor (Table 1).

The NA and the SI subscales of Persian version of the 14-item Type D have good test–retest stability over a 2-month period (test–retest r = 0.86/0.77 respectively) and the high internal consistency of Cronbach's alpha coefficient has been found to be 0.84 in patients’ group and 0.87 in healthy group for the NA subscale and 0.86 in patients’ group and 0.75 in healthy group for the SI sub-scale. The construct validity of NA and SI subscales were confirmed against neuroticism (r = 0.65) and extroversion (r = -0.62) subscales of Eysenck’s questionnaire respectively.

## Discussion

This study was performed to provide and assess the psychometric properties of the Persian version of the DS14, one of the most widely used instruments to measure NA and SI. The Persian version of the DS14 proved to be acceptable to patients and healthy people and also the present findings support the use of the DS14 to assess the traits of Type D personality in epidemiologic and clinical research.

This study indicated that 35.8% of post-MI patients and 24.6% of healthy people were classified as Type D personality. The findings proved that the Persian version of the DS14 is a reliable and valid scale to detect Type D personality. Moreover, the present results confirmed that the internal consistency of NA sub-scale and the SI sub-scale are high (coefficient alpha of 0.84 and 0.87 for the NA sub-scale and 0.86 and 0.75 for the SI sub-scale).

The construct validity of NA and SI subscales were confirmed against neuroticism and extroversion subscales of Eysenck’s questionnaire. Scores on the NA and SI scales were stable over a 2-month period of time.

The overall pattern of results was approximately close to the findings yielded in Denollet`s study about psychometric properties of the DS14 (1) and Pedersen and Denollet`s study about validity of the Type D personality construct in Danish post-MI patients (2).

Personality refers to a complex organization of the patterns of thoughts, feelings and behaviors, consistently exhibited by an individual over a long period of time (15). In other words, personality traits may be able to explain individual differences in many situations such as distress. It is important to note that while the DS14 is stable over time, NA and SI do not cover the entire range of individual differences in personality. These global traits reflect the major domains of personality in people. Individuals who are high in NA have increased vulnerability to anxiety and depression (12) and those who are high in SI have increased vulnerability to interpersonal stress and failure to adapt (16). Thus, some Type D individuals will display subclinical levels of emotional distress during their lives (17).

**Table 1:** Factor ctructure and internal consistency of persian version of DS14

Items of the DS14	MI Patients			Normal People		
	Factor I	Factor II	Internal Consistency	Factor I	Factor II	Internal Consistency
Negative Affectivity						
(2) I often make a fuss about unimportant things	0.65	0.15	0.63	0.59	0.82	0.85
(4) I often feel unhappy	0.76	0.43	0.77	0.79	0.35	0.83
(5) I am often irritated	0.76	0.29	0.74	0.79	0.22	0.84
(7) I take a gloomy view of things	0.66	0.40	0.66	0.73	0.45	0.83
(9) I am often in a bad mood	0.71	0.59	0.75	0.70	0.48	0.83
(12) I often find myself worrying about something	0.72	0.38	0.73	0.73	0.20	0.84
(13) I am often down in the dumps	0.69	0.37	0.71	0.79	0.22	0.84
Eigenvalue I for Patients = 6.101 Eigenvalue I for Normal = 5.243			α = 0.84			α = 0.87
Social Inhibition						
(1) I make contact easily when I meet people	0.29	0.72	0.70	0.14	0.74	0.85
(3) I often talk to strangers	0.14	0.74	0.69	0.38	0.52	0.86
(6) I often feel inhibited in social interactions	0.57	0.71	0.77	0.39	0.69	0.84
(8) I find it hard to start a conversation	0.42	0.75	0.75	0.28	0.78	0.84
(10) I am a closed kind of person	0.55	0.80	0.83	0.25	0.68	0.86
(11) I would rather keep other people at a distance	0.45	0.69	0.72	0.39	0.59	0.84
(14) When socializing, I don’t find the right things to talk about	0.35	0.73	0.72	0.33	0.73	0.84
Eigenvalue II for Patients = 1.63 Eigenvalue II for Normal = 1. 99			α = 0.86			α = 0.75

The DS14 NA scale evaluates the tendency to experience negative emotions including depressed mood, anxiety, anger, and hostile feelings across time/situations (1, 6). Individuals with high-NA score tend to experience sadness, tend to be anxious, and are easily irritated. The way those people deal with these emotions during social interactions may be as important as the experience of negative emotions (6). Thus, social inhibition is another detrimental factor of stress-related disease (1). Individuals with high-SI tend to inhibit the expression of these negative feelings as a strategy to cop with emotion (1, 21). The interaction of NA (which is closely related to neuroticism) and SI define Type D personality. NA and SI are 2 global traits (1) that are relevant to coronary heart disease (CHD) (5,6,22). Denollet conceptualized Type D personality and designed the DS14 to identify the people are high risk for CHD.

## Conclusion

The validity of the Type D construct was confirmed in two Iranian samples of post-MI patients and healthy people. Although the present study suggests that the Persian version of DS14 is a useful instrument in screening Type D personality in post-MI patients and healthy people, more investigations should be performed to ascertain whether diagnosis of Type D personality by the Persian version of DS14 with cutoff scores of 10 on both subscales will be confirmed by other measures.

Two limitations of this study should be mentioned here. First, psychological conditions after MI such as the depressed mood might affect the participants` answers to DS14 in patients’ group while the main purpose of this study was to examine efficiency of the Persian version of DS14 in screening Type D personality. Secondly, this study included a sample of MI patients and a limited sample of healthy people assessed by the Persian version of DS14. Thus, the findings should be considered in the context of these limitations.

## Notes

**Declaration of Interest:** None.
